# Stabilization of cervical stiffness associated with cervical pessary placement in women at risk for preterm birth

**DOI:** 10.1371/journal.pone.0359271

**Published:** 2026-09-28

**Authors:** Ioannis Kyvernitakis, Philien Lauer, Laura Bernardi, Sabrina Badir, Franz Bahlmann, Holger Maul, Maciej Osinski

**Affiliations:** 1 Department of Obstetrics and Prenatal Medicine, Asklepios Klinik Barmbek, Hamburg Germany; 2 Department of Obstetrics and Gynecology, University Hospital Kiel, Kiel Germany; 3 Pregnolia AG, Schlieren, Switzerland; 4 Department of Obstetrics and Gynecology, Buergerhospital and Clementine Children’s Hospital Frankfurt, Frankfurt, Germany; Ruijin Hospital, Shanghai Jiao Tong University School of Medicine, CHINA

## Abstract

**Purpose:**

Shortening of the cervix is a predictor of spontaneous preterm birth (PTB). It is assumed that a cervical pessary reduces mechanical stress on the internal os and consequently decreases the risk of premature delivery. The primary aim of this study was to compare longitudinal changes in cervical stiffness between women with cervical shortening (a cervical length ≤ 25 mm according to the ISUOG guidelines) treated with a cervical pessary and healthy controls.

**Methods:**

A total of 89 pregnant participants were analysed in this prospective observational study. Among them, 30 received a cervical pessary because of cervical shortening below 25 mm. The remaining 59 women served as the control group. Cervical stiffness was measured with the Pregnolia® system at two time points: between 18 + 0 and 24 + 6 weeks of gestation (first appointment) and again 4 weeks later. In the pessary cohort, the baseline measurement was obtained prior to pessary insertion.

**Results:**

At four weeks, women in the control group demonstrated a decrease in the median Cervical Stiffness Index (CSI) value: 60.8 vs. 46.7 mbar (nominal p = 0.03) although this difference did not remain statistically significant after FDR adjustment (p = 0.09). In contrast, median CSI values remained stable in the pessary group (71.2 vs. 73.7 mbar; p = 0.88). Cervical length and uterocervical angle (UCA) did not change within the observation period in either group. Moreover, the median birth weight in the normal cohort and in the Pessary cohort was 3380 g (3175–3570) and 3185 g (3011–3450), respectively (p = 0.062).

**Conclusion:**

In women with the cervical shortening treated with the pessary cervical stiffness remained stable, whereas a decline was observed in the control group. These findings support a potential association between cervical pessary treatment and stabilisation of cervical stiffness. Further studies, including untreated women with cervical shortening are needed to confirm these findings and to better understand the underlying biomechanical mechanisms.

## Introduction

The cervix plays a central role in maintaining pregnancy by providing mechanical support and an effective barrier between the intrauterine environment and the lower genital tract, thereby protecting the fetus from ascending infection and premature expulsion [1]. During normal gestation, biochemical and inflammatory processes driven by maternal–fetal signaling and fetoplacental maturation—characterized by increased prostaglandin activity—lead to progressive cervical softening and remodelling, which are essential for term parturition [1, 2]. Premature activation of these processes may result in preterm birth, which remains a major global health concern, affecting approximately 9.9% of live births worldwide [3, 4].

Spontaneous preterm birth (sPTB) is a leading cause of neonatal morbidity and mortality and is associated with significant long-term sequelae. Despite advances in perinatal care, strategies for predicting and preventing sPTB remain limited [5]. While preterm birth has a heterogeneous aetiology, cervical dysfunction is widely regarded as a key contributor. The premature onset of cervical softening, shortening, funnelling, and dilation—collectively termed precocious cervical ripening (PCCR)—is increasingly recognized as a final common pathway leading to sPTB [6–9].

Transvaginal ultrasound measurement of cervical length is a well-established method for sPTB risk stratification. ISUOG recommends routine assessment between 18 and 24 weeks’ gestation, with a cervical length ≤ 25 mm indicating increased risk [10]. However, cervical length reflects only one aspect of cervical remodelling and does not capture changes in tissue consistency. (2) Cervical softening, a key component of PCCR, has traditionally been assessed subjectively, limiting reproducibility and clinical utility [11]. Emerging evidence suggests that cervical integrity exists along a continuum influenced by both structural and biomechanical factors, highlighting the need for objective *in vivo* assessment tools [12, 13].

The Arabin cervical pessary represents a non-invasive intervention for women with cervical shortening, with outcomes in some studies comparable to cervical cerclage [14]. Subgroup analyses suggest greater benefit in women without funnelling and with a narrow uterocervical angle [15]. Beyond prolonging gestation, pessary therapy may influence cervical biomechanics; however, data on its effect on cervical stiffness remain limited. Novel technologies, such as the Pregnolia® system, now enable objective, reproducible assessment of cervical stiffness *in vivo*, offering new insights into the biomechanical effects of pessary treatment [16]. Further validation in adequately powered randomized controlled trials is required [16, 17].

This study evaluates whether pessary therapy in women with cervical shortening stabilizes cervical stiffness over time, providing mechanistic insight into its potential role as an early, non-invasive intervention to reduce the risk of spontaneous preterm birth.

## Materials and methods

### Study design and setting

This prospective, non-interventional, post-market, monocentric longitudinal cohort study was conducted at a tertiary maternity teaching hospital (Asklepios Klinik Hamburg-Barmbek). The aim was to evaluate the effect of cervical pessary treatment on cervical biomechanical properties in pregnant women with cervical shortening and to compare these findings with those from a gestational age–matched low-risk control cohort.

The study was approved by the Ethics Committee of the Hamburg Medical Council (approval number 2021–100680-BO-ff; 1 November 2021). Written informed consent was obtained from all participants.

Participant recruitment: Participants were prospectively recruited between 23 November 2021 and 26 June 2024. Written informed consent was obtained from all participants before enrollment in the study. The study protocol was approved by the appropriate institutional ethics committee and was conducted in accordance with the Declaration of Helsinki.

Available birth outcome data were collected until 31 December 2024. During data collection and analysis, the authors had access to information that could identify individual participants.

The study protocol, feasibility assessment, and participant eligibility criteria have been published previously; however, no study outcomes or Cervical Stiffness Index (CSI) data from the present cohort were reported in that publication (3).

### Study population

A total of 98 women with singleton pregnancies were recruited between 18 + 0 and 24 + 6 weeks’ gestation. The study group comprised 34 women treated with an Arabin cervical pessary because of a short cervix, defined as a cervical length ≤ 25 mm according to the ISUOG guidelines. The control group included 64 asymptomatic women with low-risk singleton pregnancies, normal cervical length, and no risk factors for preterm birth.

For analyses requiring available birth and follow-up data, 89 participants were included: 59 women in the control cohort and 30 women in the study cohort. As not all measurements were available for all participants at both study visits, the number of participants varied between individual analyses according to data availability. Among women in the control cohort, baseline CSI measurements were available for 58 of 59 participants, as one participant had missing baseline CSI data. The corresponding sample size is reported for each analysis where applicable.

### Study procedures

Each participant attended two study visits approximately four weeks apart. The first appointment occurred between 18 + 0 and 24 + 6 weeks’ gestation and the follow-up measurement approximately 4 weeks later. At each visit, participants underwent a speculum examination to confirm eligibility, followed by transvaginal ultrasound (TVUS) assessment of cervical length and uterocervical angle (UCA), and cervical stiffness measurements. In the pessary group, baseline stiffness measurements were obtained prior to pessary insertion.

### Cervical stiffness assessment

Cervical stiffness was assessed using the Pregnolia® system, a CE-certified medical device designed to quantify cervical tissue stiffness objectively. The system measures the vacuum pressure required to deform ectocervical tissue by 4 mm, expressed as the Cervical Stiffness Index (CSI, mbar). Higher CSI values indicate increased tissue stiffness.

Measurements were performed according to the manufacturer’s instructions for use. After speculum placement and removal of excess mucus, the probe was applied to the anterior cervical lip at the 12 o’clock position. Three consecutive CSI measurements were obtained without time delay and recorded for analysis, and the mean value was used for the analysis. All operators received standardized training in device handling.

### Ultrasound assessment

TVUS examinations were performed in the dorsal lithotomy position with an empty bladder, following established fetal medicine guidelines. Cervical length was measured along the longitudinal cervical axis using minimal probe pressure. The uterocervical angle was measured on the image demonstrating the shortest cervical length, defined as the angle between a line connecting the internal and external os and a line parallel to the lower anterior uterine wall.

### Study outcomes

The primary outcome was the absolute CSI value and its change over four weeks in pessary-treated women compared with controls. Secondary outcomes included associations between baseline CSI, longitudinal changes in CSI, and pregnancy outcomes, including gestational age at delivery.

### Statistical analysis

Statistical analyses were performed using R software (version 4.4.1; R Foundation for Statistical Computing, Vienna, Austria). Continuous variables were summarized as medians and interquartile ranges (IQR), unless otherwise stated, and categorical variables as counts and percentages.

Between-group comparisons of continuous variables were performed using the Wilcoxon rank-sum test, while categorical variables were compared using Fisher’s exact test. Associations between continuous variables were assessed using linear regression models, including multivariable linear regression where appropriate.

As not all participants had CSI measurements available at both study visits, the primary comparisons between visit 1 and visit 2 were performed using all available CSI measurements at each visit, using the Wilcoxon rank-sum test. Thus, participants who had a CSI measurement at visit 1 but not at visit 2 were included in the visit 1 group and contributed to the unpaired comparison, but were not included in the visit 2 group. As a sensitivity analysis, longitudinal changes in CSI were additionally assessed using the paired Wilcoxon signed-rank test among participants with CSI measurements available at both visits, first restricted to participants with available delivery data and subsequently including all participants irrespective of delivery-data availability.

To account for multiple comparisons within prespecified outcome sets, p-values were adjusted using the false discovery rate (FDR) approach according to the Benjamini–Hochberg procedure. Unless otherwise specified, p-values reported in the text are nominal (unadjusted) p-values; FDR-adjusted p-values are reported where multiple-comparison correction was applied. FDR correction was applied separately to predefined sets of related comparisons, including the overall, term, and preterm comparisons at each study visit and to the corresponding set of longitudinal comparisons across cohorts.

### Follow-up and data collection

Delivery and neonatal outcome data were obtained from medical records within 4–8 weeks postpartum. Collected data included gestational age at delivery, mode and onset of labour, neonatal birth weight, neonatal morbidity, maternal hospitalization, and neonatal intensive care unit admission.

### Sample size

Formal sample size calculation was not performed due to the exploratory nature of the study and limited prior data on cervical stiffness assessment using the Pregnolia® system in pregnancies affected by precocious cervical ripening. Consequently, the study may be underpowered to detect small-to-moderate between-group differences, and results should be interpreted as exploratory rather than confirmatory.

## Results

A total of 98 women were enrolled in the study, including 64 in the control cohort and 34 in the pessary cohort. Birth outcome and follow-up data were unavailable for nine participants due to loss to follow-up. Therefore, the final analysis included 89 women with available birth outcome and follow-up data, comprising 59 women in the control cohort and 30 women in the pessary cohort.

Among the women in the control cohort, CSI measurements at the first visit were unavailable for one participant, resulting in 58 women available for the baseline CSI analysis. Overall, CSI data were available for 58 women in the control cohort at the first visit and 45 at the second visit; the missing second-visit measurements were due to loss to follow-up or failure to attend the scheduled follow-up visit.

In the pessary cohort, CSI data were available for 30 women at the first visit and 25 at the second visit; the missing second-visit measurements were likewise due to loss to follow-up or failure to attend the scheduled follow-up visit.

Baseline demographics were comparable between groups regarding age, height, gravidity, parity, and obstetric history (Table 1).

**Table 1:** Demographic characteristics of the included patients, stratified by cohort.

Characteristic	Normal cohort N = 59^1^	Pessary cohort N = 30^1^	p-value^2^
Ethnicity			0.12
African	4 (6.8%)	1 (3.3%)	
Asian	4 (6.8%)	7 (23%)	
Caucasian	42 (71%)	20 (67%)	
Other	9 (15%)	2 (6.7%)	
Age	34.0 (29.0, 38.0)	36.0 (32.5, 39.0)	0.10
Height	166 (161, 170)	168 (163, 174)	0.095
Gravidity			0.11
I	26 (44%)	8 (27%)	
II	9 (15%)	4 (13%)	
III	9 (15%)	7 (23%)	
IV	7 (12%)	3 (10%)	
V	6 (10%)	2 (6.7%)	
VI	1 (1.7%)	4 (13%)	
VII	0 (0%)	0 (0%)	
VIII	0 (0%)	2 (6.7%)	
IX	1 (1.7%)	0 (0%)	
Parity			0.4
0	33 (56%)	14 (47%)	
1	14 (24%)	6 (20%)	
2	9 (15%)	8 (27%)	
3	3 (5.1%)	1 (3.3%)	
4	0 (0%)	1 (3.3%)	
Prior vaginal delivery			0.072
No	41 (69%)	15 (50%)	
Yes	18 (31%)	15 (50%)	
History of medical/chronic disease	18 (32%)	8 (28%)	0.7
Unknown	2	1	
Prior LLETZ	3 (5.1%)	2 (6.7%)	>0.9
No prior cone biopsy	59 (100%)	30 (100%)	n/a

^1^n (%); Median (IQR)

^2^Fisher’s exact test for categorical variables; Wilcoxon rank-sum test for continuous variables; Pearson’s chi-squared test when applicable.

Clinical outcomes and biomechanical parameters, including gestational age at delivery, preterm birth rate, and birth weight, are presented per cohort in Table 2.

**Table 2:** Clinical outcomes and biomechanical parameters in the control and pessary cohorts. Outcomes are reported for the overall population as well as stratified by visit (baseline and follow-up). Preterm birth (PTB) was defined as delivery before 37 weeks of gestation. Missing data are indicated as “Unknown”.

Outcome	Normal cohort N=59^1^	95% CI^2^	Pessary cohort N=30^1^	95% CI^2^	p-value^3^
GA at delivery	39.40 (38.45, 40.40)	39, 40	38.10 (37.15, 39.38)	36, 39	0.005
PTB < 37 weeks	5 (8.5%)	3.2%, 19%	5 (17%)	6.3%, 35%	0.3
Birth weight	3,380 (3,175, 3,570)	3,166, 3,452	3,185 (3,011, 3,450)	2,817, 3,358	0.062
**Visit 1 (baseline)**					
GA at measurement	21.90 (20.65, 22.85)	21, 22	21.55 (20.90, 23.38)	21, 22	0.8
CSI mean (mbar)	61 (44, 82)	58, 74	71 (52, 138)	71, 113	0.087
Unknown	1		0		
CL (mm)	41 (35, 44)	38, 42	23 (21, 26)	21, 24	<0.001
UCA (°)	90 (87, 95)	90, 93	91 (88, 98)	91, 96	0.3
Discomfort related to CSI measurement [0 – 100]	10 (0, 20)	8.6, 17	10 (0, 20)	8.7, 19	0.5
**Visit 2 (follow-up)**					
GA at measurement	25.90 (24.30, 27.85)	25, 27	26.95 (25.33, 27.40)	26, 27	0.2
Unknown	12		0		
CSI mean (mbar)	47 (29, 78)	45, 64	74 (44, 134)	68, 108	0.004
Unknown	14		5		
CL (mm)	36 (32, 44)	35, 39	24 (20, 27)	20, 26	<0.001
Unknown	12		1		
UCA (°)	93 (87, 100)	92, 96	97 (90, 100)	93, 99	0.2
Unknown	13		1		
Discomfort related to CSI measurement [0 – 100]	10 (0, 16)	6.2, 15	15 (10, 30)	11, 21	0.062
Unknown	27		5		

^1^n (%); Median (IQR).

^2^95% Confidence Interval.

^3^Fisher’s exact test for categorical variables; Wilcoxon rank-sum test for continuous variables; Pearson’s chi-squared test when applicable.

### Primary Outcome – Cervical Stiffness Index (CSI)

After four weeks, women in the control group exhibited a statistically significant decrease in median Cervical Stiffness Index (CSI) values 60.8 vs. 46.7 (p = 0.03; Fig 1A). However, this difference did not remain statistically significant after adjustment for multiple comparisons using the false discovery rate (FDR) method (p = 0.09). In contrast, the pessary group showed no statistically significant change in CSI over the same period (71.2 vs. 73.7, p = 0.88; Fig 1B).

**Fig 1:**
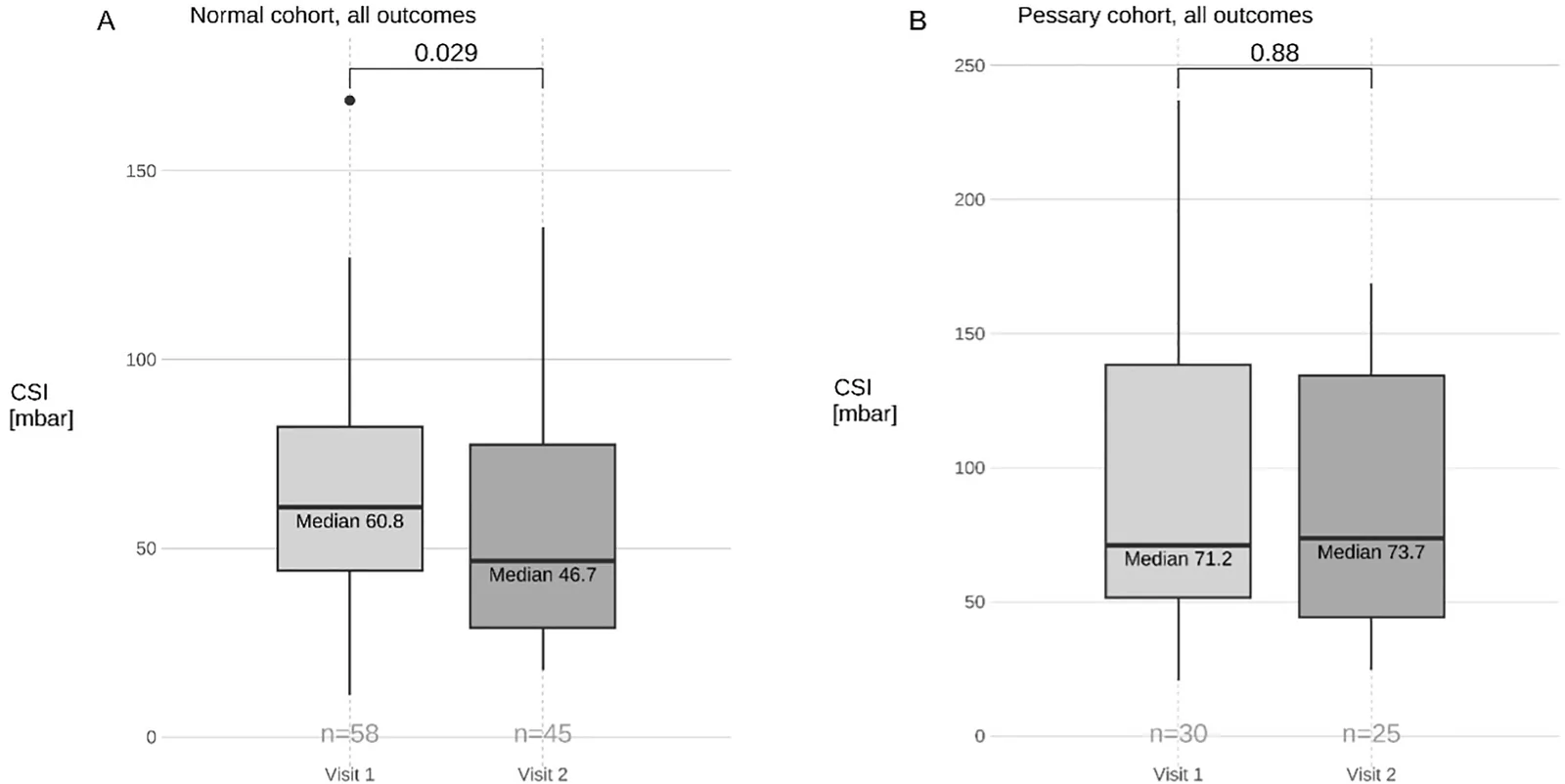
Cervical Stiffness Index Values in the PCCR – Pessary and in Normal Cohort throughout the second and third trimester. Legend: Box-and-whisker plots showing Cervical Stiffness Index (CSI, mbar) values at baseline and follow-up in the control cohort and the pessary cohort. **A:** In the control cohort, median CSI decreased from 60.8 mbar at baseline to 46.7 mbar at follow-up, indicating physiological cervical softening over time. **B:** In the pessary cohort, median CSI remained stable from 71.2 mbar at baseline to 73.7 mbar at follow-up, suggesting preservation of cervical stiffness after pessary placement. Boxes represent the interquartile range, horizontal lines indicate medians, and whiskers represent data variability. P-values indicate within-group comparisons between visits. CSI = Cervical Stiffness Index (mbar).

As a sensitivity analysis, longitudinal changes in CSI were assessed using a paired Wilcoxon signed-rank test among participants with CSI measurements available at both visits and available delivery data. Forty-five control and 25 pessary participants contributed to this analysis. In the control cohort, CSI decreased between visits (nominal p = 0.019; FDR-adjusted p = 0.057), whereas no significant change was observed in the pessary cohort (p = 0.737; FDR-adjusted p = 0.737). When all participants with CSI measurements available at both visits were included, irrespective of availability of delivery data, 46 control and 26 pessary participants contributed to the paired analysis. The corresponding p-values were 0.028 (FDR-adjusted p = 0.057) in the control cohort and 0.517 (FDR-adjusted p = 0.690) in the pessary cohort.

Interestingly, at visit 2, median CSI values were significantly lower in the control group compared with the pessary group (46.7 vs 73.7 mbar, n = 45 and n = 25, respectively, p = 0.004; Table 2), proving a stabilising effect of pessary therapy on cervical biomechanical properties. This between-group difference remained statistically significant after FDR correction (p = 0.03).

These group-level effects were further supported by non-parametric testing. A Kruskal-Wallis test revealed an overall difference in CSI across the four subgroups (control visit 1, n = 58; control visit 2, n = 45; pessary visit 1, n = 30; pessary visit 2, n = 25; p = 0.002). Post hoc analysis (Dunn’s test) identified significant difference between the control group at follow-up and the pessary group at baseline (adjusted p = 0.005), and between the control group at follow-up and the pessary group at follow-up (adjusted p = 0.019). These findings are consistent with the observed between-group differences in CSI at follow-up.

### Cervical Length (CL)

Cervical length was significantly shorter in the pessary cohort than in the control cohort at both visits (visit 1: n = 59 and 30; visit 2: n = 47 and 29, respectively; all p < 0.001), reflecting the treatment indication based on cervical shortening. These between-group differences remained statistically significant after correction for multiple comparisons using the FDR method.

Within each cohort, cervical length remained stable over time, with no significant change from baseline to follow-up in either the control cohort (41–36 mm; n = 59 and 47; p = 0.083) or the pessary cohort (23–24 mm; n = 30 and 29; p = 0.77).

### Uterocervical Angle (UCA)

There was no statistically significant change in UCA between visits in either cohort (control: n = 59 and 46; pessary: n = 30 and 29).

### Multiple linear regression

To explore which clinical or sonographic parameters associated with CSI at baseline (visit 1), multiple linear regression was performed in 94 participants with complete data for all variables included in the model. In the multivariable model including cervical length, cohort allocation, and prior vaginal delivery, only prior vaginal delivery was significantly associated with lower CSI values (p = < 0.001). Women with a history of vaginal delivery had lower CSI values at baseline, which may reflect prior structural or biomechanical cervical changes. Cervical length and group allocation were not significantly associated with CSI in the multivariable model.

To further assess potential confounding of longitudinal CSI changes, adjusted linear regression analyses were performed among women with CSI measurements available at both visits (n = 72). Follow-up CSI was significantly associated with baseline CSI (p < 0.001), whereas cervical length, prior vaginal delivery, and cohort allocation were not significantly associated with follow-up CSI. Similarly, when analysing the change in CSI between visits, higher baseline CSI was associated with a greater decrease in CSI over follow-up (p < 0.001), while cervical length, prior vaginal delivery, and cohort allocation were not significantly associated with CSI change.

### Pain and Discomfort

Mean pain and discomfort scores for the CSI measurement were low in both groups at baseline (control: 12.4 ± 15.8, n = 64; pessary: 14.3 ± 13.9, n = 31; p = 0.33). At follow-up, scores remained low, with mean values of 11.1 ± 12.0 (n = 33) and 16.5 ± 12.0 (n = 26), respectively (p = 0.064). Pessary insertion was associated with slightly higher discomfort than CSI measurement alone, with a mean score of 18.4 ± 12.7 (n = 31).

These analyses were performed using all participants with available discomfort scores at each time point, regardless of subsequent loss to follow-up or missing outcome data in other study variables. Thus, the results slightly differ from the ones reported in Table 2, for which only women with outcome data are reported.

## Discussion

Our results demonstrate that pessary placement not only stabilises cervical length but also suggests a possible attenuation of the physiological decline in cervical stiffness typically observed in the second trimester of pregnancy. Women in the control group showed a decline in median CSI values over time, whereas values remained stable in the pessary group, supporting a potential stabilising effect of pessary therapy on cervical biomechanical properties.

These findings also suggest that the pessary may exert a protective mechanical influence on the internal os, thereby reducing cervical deformation and delaying tissue softening.

This study adds a novel dimension to the understanding of how pessary treatment may work. While previous research has mainly focused on geometric sacralisation of the cervix, our findings introduce the preservation of cervical stiffness as a potential key mechanism of action [4].

This aligns with the concept of preventing premature cervical remodelling — a central feature in the pathophysiology of both cervical shortening and cervical insufficiency — that has so far lacked real-time biomechanical evidence.

The cervical pessary, a flexible silicone medical device, has been in use since 1959, originally introduced as a treatment for recurrent miscarriage attributed to cervical insufficiency [5 18]. Although its precise mode of action remains unclear, it is widely hypothesised that the device alleviates mechanical strain on the internal os by altering the angle of the cervical canal and redistributing the intrauterine pressure more evenly across the cervix [19].

In addition to stabilising the cervix, the pessary may induce direct mechanical compression of cervical tissue, particularly when the inner diameter of the pessary is smaller than the outer diameter of the cervix. This compression alters the loading state of cervical tissues, with unknown biological consequences. Biomechanical modelling further suggests that when the cervical canal is well-aligned with the longitudinal uterine axis, stress on the internal os is minimised. In contrast, a posteriorly angled cervix experiences greater tensile strain at the internal os without meaningful reduction in axial load, potentially contributing to cervical dysfunction [20].

The observed stabilization of CSI in the pessary group may reflect a modulation of the distension process at the internal os, which plays a central role in cervical failure. From a clinical perspective, these findings support the timely identification and management of women with cervical shortening and PCCR Spectrum. This unifying concept has emerged to describe the continuum between cervical insufficiency, according to ACOG defined as painless mid-trimester dilation without contractions [6] — and cervical shortening, which is primarily diagnosed sonographically (CL < 25 mm before 24 weeks) in asymptomatic women [7]. Transvaginal sonographic measurement of cervical length is an established method for identifying women at increased risk of spontaneous preterm birth (2).

Cervical insufficiency is commonly defined as the inability of the cervix to maintain closure throughout pregnancy, either due to intrinsic structural weakness or because of acquired compromise. RCOG states that it remains largely a diagnosis based on clinical history rather than on universally established objective criteria. Furthermore, emerging evidence suggests that cervical integrity exists along a continuum, influenced not only by the intrinsic structural properties of the cervix but also by dynamic functional mechanisms involved in premature effacement and dilatation [8].

Cervical insufficiency and cervical shortening represent distinct entities with overlapping features, but their differentiation in clinical practice is often challenging. Moreover, this distinction may be of limited relevance for therapeutic decision-making, as both conditions share similar management strategies, including vaginal progesterone, cerclage, and pessary (AWMF Guideline 015–025, 2024, Germany, Switzerland and Austria) [9,10]. Emphasised by ACOG – given that the diagnosis of cervical insufficiency is frequently established retrospectively [6], the PCCR spectrum provides a pragmatic framework that may help clinicians intervene earlier and more effectively.

The concept of the PCCR enables a more inclusive and functional classification of patients with morphologic or biomechanical cervical vulnerability. It integrates both functional aspects (e.g., painless dilation) and measurable morphological markers (e.g., CL < 25 mm) and may thus improve clinical decision-making and research design.

Our findings also highlight the limitations of current guidelines, which primarily focus on cervical length as the only mechanical parameter for risk stratification. The German S2k guideline supports the use of the pessary in women with a shortened cervix but does not yet consider cervical stiffness as a measurable or targetable clinical marker (AWMF Guideline 015–025, 2024) [9,10].

Although promising in principle, elastographic techniques have proven to be unreliable due to variability and inconsistent reproducibility across operators and devices [11,12].

In contrast, the Pregnolia System, based on a standardised aspiration technique, has demonstrated high intra- and interobserver reliability (<3%) and is suitable for longitudinal monitoring. From a mechanistic standpoint, the preservation of cervical stiffness may reflect a dampening of distension forces at the internal os — particularly when pessary placement leads to improved uterocervical alignment. Recent work suggests that a more acute uterocervical angle reduces tensile strain at the internal os and may therefore protect against early cervical remodelling [13,14].

Given the reproducible differences in CSI observed in our study, we suggest integrating cervical stiffness assessments into future research protocols and potentially into clinical practice, pending standardization and broader validation of devices such as Pregnolia® [15].

The System, enables quantitative, objective measurement of cervical stiffness using an aspiration based technique [16]. Clinical and in vitro studies have demonstrated its high intra- and inter-observer reliability (≤ 3% variability) compared to traditional digital palpation. Notably, data indicate that the cervix undergoes softening several weeks before morphological shortening is observed via ultrasound. Ongoing feasibility trials (e.g., “PRECISION,” CASPAR, STIPP) are further evaluating its predictive value for spontaneous preterm birth in high-risk and induction-of-labour cohorts [16].

Colacourci et al. also demonstrated clearly, a softer cervix is associated with an increased risk of sPTB. Breuking et al. showed an association of reduced cervical stiffness with an increased risk of sPTB within 7 days in patients with prodromal symptoms of threatened sPTB [17]. Hösli et. al. demonstrated that CSI assessment is safe. In this study the discomfort for the pregnant women was minimal, moreover the authors stated that women experiencing a sPTB have a significantly softer cervix than women giving birth at term [21].

Stone and House reported significantly lower cervical stiffness in women requiring ultrasound-indicated cerclage compared with healthy controls using the aspiration-based device, supporting the potential of aspiration-based technology as an objective tool for assessing cervical biomechanical properties during pregnancy [22].

Furthermore, incorporating stiffness into predictive models may improve individual risk assessment, particularly in cases of idiopathic cervical shortening, where no classical risk factors or biochemical alterations are present [23,24]. Identifying such cases earlier through biomechanical evaluation could improve patient selection for interventions, reduce unnecessary treatment, and contribute to more personalized care.

Cervical shortening is one of the most common pathways to spontaneous preterm birth and the measurement of cervical length via transvaginal ultrasound between 16 and 24 weeks’ gestation is well established as the gold standard for preterm birth risk assessment [7,25]. However, it provides only a morphological dimension. Our data suggest that biomechanical assessment, in the form of cervical stiffness measurement, may complement sonographic findings and enhance early risk stratification.

### Limitations of the study

This study has several limitations. First, the control group consisted of low-risk women with normal cervical length, whereas the pessary group included women with cervical shortening. Therefore, the two cohorts were not directly comparable. Second, because no untreated short-cervix group was available, the observational study design does not permit causal inference regarding the effect of cervical pessary placement on cervical stiffness. In our institution, cervical pessary placement is part of routine clinical management for women with a short cervix, making the inclusion of an untreated comparison group impractical, and therefore the project of the study did not design the comparison with group of women with a short cervix who did not receive a pessary, so we were not able to examine CSI values of natural course of cervical remodeling in high-risk pregnancies.

Finally, the relatively small sample size may have limited the statistical power to detect subtle longitudinal changes. Moreover, the study was not designed or powered to assess clinically relevant outcomes such as spontaneous preterm birth. Therefore, the observed stabilization of cervical stiffness under pessary treatment should be interpreted as exploratory and hypothesis-generating rather than clinically definitive.

### Summary

In summary, our study is the first to report that cervical stiffness remained stable over time in women with mid-trimester cervical shortening treated with a cervical pessary. These findings support a potential association between cervical pessary treatment and preservation of cervical stiffness. Further studies, including untreated women with cervical shortening are needed to confirm these findings and to better understand the underlying biomechanical mechanisms.

## References

[1] Myers KM, Feltovich H, Mazza E, Vink J, Bajka M, Wapner RJ, et al. The mechanical role of the cervix in pregnancy. J Biomech. 2015;48(9):1511–23. doi: 10.1016/j.jbiomech.2015.02.065 25841293 PMC4459908

[2] Kagan KO, To M, Tsoi E, Nicolaides KH. Preterm birth: the value of sonographic measurement of cervical length. BJOG. 2006;113 Suppl 3:52–6. doi: 10.1111/j.1471-0528.2006.01124.x 17206965

[3] Kyvernitakis I, Lauer P, Malan M, Badir S, Maul H. A novel aspiration technique to assess cervical remodelling in patients with or without cervical shortening: Sequence of first changes, definition of cut-off values and impact of cervical pessary, stratified for cervical length. PLoS One. 2023;18(4):e0283944. doi: 10.1371/journal.pone.0283944 37023066 PMC10079006

[4] Kyvernitakis I, Baschat AA, Malan M, Rath W, Berger R, Henrich W, et al. Cervical pessary to prevent preterm birth and poor neonatal outcome: An integrity meta-analysis of randomized controlled trials focusing on adherence to the European Medical Device Regulation. Int J Gynaecol Obstet. 2024;165(2):607–20. doi: 10.1002/ijgo.15169 37830250

[5] Kyvernitakis I, Maul H, Bahlmann F. Controversies about the Secondary Prevention of Spontaneous Preterm Birth. Geburtshilfe Frauenheilkd. 2018;78(6):585–95. doi: 10.1055/a-0611-5337 29962517 PMC6018068

[6] ACOG Practice Bulletin No.142: Cerclage for the management of cervical insufficiency. Obstet Gynecol. 2014;123(2 Pt 1):372–9. doi: 10.1097/01.AOG.0000443276.68274.cc 24451674

[7] Coutinho CM, Sotiriadis A, Odibo A, Khalil A, D’Antonio F, Feltovich H, et al. ISUOG practice guidelines: Role of ultrasound in the prediction of spontaneous preterm birth. Ultrasound Obstet Gynecol. 2022;60(3):435–56. doi: 10.1002/uog.26020 35904371

[8] Shennan A, Story L, Royal College of Obstetricians and Gynaecologists. Cervical cerclage. BJOG: An International Journal of Obstetrics & Gynaecology. 2022;129(7):1178–210. doi: 10.1111/1471-0528.17003 35199905

[9] Berger R, Abele H, Bahlmann F, Bedei I, Doubek K, Felderhoff-Müser U, et al. Prevention and therapy of preterm birth. Guideline of the DGGG, OEGGG and SGGG (S2k level, AWMF registry number 015/025, February 2019) - part 2 with recommendations on the tertiary prevention of preterm birth and the management of preterm premature rupture of membranes. Z Geburtshilfe Neonatol. 2019;223(6):6. doi: 10.1055/a-1008-8730 31423017 PMC6690742

[10] Berger R, Abele H, Bahlmann F, Bedei I, Doubek K, Felderhoff-Müser U, et al. Prevention and therapy of preterm birth. Guideline of the DGGG, OEGGG and SGGG (S2k level, AWMF registry number 015/025, February 2019) - part 2 with recommendations on the tertiary prevention of preterm birth and the management of preterm premature rupture of membranes. Geburtshilfe Frauenheilkd. 2019;79(8):8. doi: 10.1055/a-0903-2735 31423017 PMC6690742

[11] Evaluation of cervical elastography for prediction of spontaneous preterm birth in low-risk women: a prospective study. https://pubmed.ncbi.nlm.nih.gov/31626344/. Accessed 2025 June 12. 10.1002/jum.15149 31626344

[12] Wozniak S, Czuczwar P, Szkodziak P, Milart P, Wozniakowska E, Paszkowski T. Elastography in predicting preterm delivery in asymptomatic, low-risk women: a prospective observational study. BMC Pregnancy Childbirth. 2014;14:238. doi: 10.1186/1471-2393-14-238 25041946 PMC4223586

[13] Dziadosz M, Bennett T-A, Dolin C, West Honart A, Pham A, Lee SS, et al. Uterocervical angle: a novel ultrasound screening tool to predict spontaneous preterm birth. Am J Obstet Gynecol. 2016;215(3):376.e1-7. doi: 10.1016/j.ajog.2016.03.033 27018466

[14] Prospective evaluation of the uterocervical angle as a predictor of spontaneous preterm birth. https://pubmed.ncbi.nlm.nih.gov/32311754/. Accessed 2025 June 12. 10.1111/aogs.13879 32311754

[15] Badir S, Mazza E, Zimmermann R, Bajka M. Cervical softening occurs early in pregnancy: Characterization of cervical stiffness in 100 healthy women using the aspiration technique. Prenat Diagn. 2013;33(8):737–41. doi: 10.1002/pd.4116 23553612

[16] Breuking S, Oudijk MA, van Eekelen R, de Boer MA, Pajkrt E, Hermans F. Assessment of cervical softening and the prediction of preterm birth (STIPP): Protocol for a prospective cohort study. BMJ Open. 2023;13(11):e071597. doi: 10.1136/bmjopen-2023-071597 37989370 PMC10668305

[17] Breuking SH, Oudijk MA, Gravesteijn BY, de Boer MA, Pajkrt E, Hermans FJR. Assessment of cervical softening and the prediction of preterm birth (STIPP study): A prospective cohort study. Am J Obstet Gynecol. 2026;234(3):e90–2. doi: 10.1016/j.ajog.2025.10.039 41192639

[18] Treatment of habitual abortion due to cervical incompetence - The Lancet [Internet]. [cited 2025 Jun 12]. Available from: https://www.thelancet.com/journals/lancet/article/PIIS0140-6736(59)92242-1/fulltext

[19] VITSKY M. Simple treatment of the incompetent cervical os. Am J Obstet Gynecol. 1961;81:1194–7. doi: 10.1016/s0002-9378(15)33352-4 13781669

[20] Koullali B, Westervelt AR, Myers KM, House MD. Prevention of preterm birth: Novel interventions for the cervix. Semin Perinatol. 2017;41(8):505–10. doi: 10.1053/j.semperi.2017.08.009 28988725 PMC5711549

[21] Hösli I, Hebisch G, Bajka M, Quack Lötscher K, Winkler A, Schäffer L, et al. Cervical stiffness in mid-pregnancy: a predictive tool using a novel aspiration device for spontaneous preterm birth risk assessment. Am J Obstet Gynecol MFM. 2026;8(2):101867. doi: 10.1016/j.ajogmf.2025.101867 41354384

[22] Stone J, House M. Measurement of cervical softness before cerclage placement with an aspiration-based device. Am J Obstet Gynecol MFM. 2023;5(4):100881. doi: 10.1016/j.ajogmf.2023.100881 36724813

[23] Feng Q, Duan H, Ju X, Appiah K, Yip KM, Tai Y-Y, et al. Prediction of spontaneous preterm birth by cervical length in the first trimester of pregnancy: Comparison of two measurement methods. Acta Obstet Gynecol Scand. 2021;100(7):1305–12. doi: 10.1111/aogs.14138 33683725

[24] Dahman HAB. Risk factors associated with preterm birth: a retrospective study in Mukalla Maternity and Childhood Hospital, Hadhramout Coast/Yemen. Sudan J Paediatr. 2020;20(2):99–110. doi: 10.24911/SJP.106-1575722503 32817730 PMC7423304

[25] Kagan KO, Sonek J. How to measure cervical length. Ultrasound Obstet Gynecol. 2015;45(3):358–62. doi: 10.1002/uog.14742 25632014

